# A Multi-Omics Analysis Pipeline for the Metabolic Pathway Reconstruction in the Orphan Species *Quercus ilex*

**DOI:** 10.3389/fpls.2018.00935

**Published:** 2018-07-11

**Authors:** Cristina López-Hidalgo, Victor M. Guerrero-Sánchez, Isabel Gómez-Gálvez, Rosa Sánchez-Lucas, María A. Castillejo-Sánchez, Ana M. Maldonado-Alconada, Luis Valledor, Jesus V. Jorrín-Novo

**Affiliations:** ^1^Agroforestry and Plant Biochemistry and Proteomics Research Group, Department Biochemistry and Molecular Biology, Universidad de Córdoba, Córdoba, Spain; ^2^Instituto de Agricultura Sostenible, Córdoba, Spain; ^3^Departamento de Biología de Organismos y Sistemas, Universidad de Oviedo, Oviedo, Spain

**Keywords:** *Quercus ilex*, omics, metabolome, proteome, transcriptome

## Abstract

Holm oak (*Quercus ilex*) is the most important and representative species of the Mediterranean forest and of the Spanish agrosilvo-pastoral “dehesa” ecosystem. Despite its environmental and economic interest, Holm oak is an orphan species whose biology is very little known, especially at the molecular level. In order to increase the knowledge on the chemical composition and metabolism of this tree species, the employment of a holistic and multi-omics approach, in the Systems Biology direction would be necessary. However, for orphan and recalcitrant plant species, specific analytical and bioinformatics tools have to be developed in order to obtain adequate quality and data-density before to coping with the study of its biology. By using a plant sample consisting of a pool generated by mixing equal amounts of homogenized tissue from acorn embryo, leaves, and roots, protocols for transcriptome (NGS-Illumina), proteome (shotgun LC-MS/MS), and metabolome (GC-MS) studies have been optimized. These analyses resulted in the identification of around 62629 transcripts, 2380 protein species, and 62 metabolites. Data are compared with those reported for model plant species, whose genome has been sequenced and is well annotated, including *Arabidopsis*, japonica rice, poplar, and eucalyptus. RNA and protein sequencing favored each other, increasing the number and confidence of the proteins identified and correcting erroneous RNA sequences. The integration of the large amount of data reported using bioinformatics tools allows the Holm oak metabolic network to be partially reconstructed: from the 127 metabolic pathways reported in KEGG pathway database, 123 metabolic pathways can be visualized when using the described methodology. They included: carbohydrate and energy metabolism, amino acid metabolism, lipid metabolism, nucleotide metabolism, and biosynthesis of secondary metabolites. The TCA cycle was the pathway most represented with 5 out of 10 metabolites, 6 out of 8 protein enzymes, and 8 out of 8 enzyme transcripts. On the other hand, gaps, missed pathways, included metabolism of terpenoids and polyketides and lipid metabolism. The multi-omics resource generated in this work will set the basis for ongoing and future studies, bringing the Holm oak closer to model species, to obtain a better understanding of the molecular mechanisms underlying phenotypes of interest (productive, tolerant to environmental cues, nutraceutical value) and to select elite genotypes to be used in restoration and reforestation programs, especially in a future climate change scenario.

## Introduction

Holm oak (*Quercus ilex*) is the most representative species of the Mediterranean forest, of great importance from an environmental and economic point of view (Rigo and De Caudullo, 2016). Being the key element of the Spanish agro-forestry-pastoral ecosystem “Dehesa,” its fruit, the acorn, is the basis of the staple food of the renowned “black leg” pork (Cantos et al., 2003). *Quercus* spp. have been used in the construction of wine barrels, contributing to the organoleptic properties of the maturing wine (Chira and Teissedre, 2014). The use of acorns in human nutrition and for pharmaceutical purposes has a long history. Employed in ancient civilizations, mainly in Italy and Spain, as food or beverage, nowadays it is far from being consumed like other common nuts (Rakić et al., 2006; Al-Rousan et al., 2013; Meijón et al., 2016). As a nutritionally rich product, and because of its high nutraceutical value, the interest of integrating acorns into the human diet or as a functional food has been raised (Vinha et al., 2016a; Hadidi et al., 2017).

Despite its environmental and economic interest, Holm oak is still an orphan species whose biology is almost unknown, especially at the molecular level. Nevertheless, the work of our group and others, has contributed to acquiring the knowledge on this species, focusing on natural variability (Valero-Galván et al., 2011; Akcan et al., 2017), seed germination and seedling growth (Echevarría-Zomeño et al., 2009; Romero-Rodríguez et al., 2015), physiology (Valero-Galván et al., 2012a), and biotic and abiotic stress-responses (Echevarría-Zomeño et al., 2009; Sghaier-Hammami et al., 2013; Sardans et al., 2014; Simova-Stoilova et al., 2015). The above publications, provide fragmented information, mostly derived from classical biochemical approaches and, to a much lesser extent, those of proteomics (Valero-Galván et al., 2011; Romero-Rodríguez et al., 2014, 2015) transcriptomics (Guerrero-Sanchez et al., 2017), or metabolomics (Rakić et al., 2006; Rabhi et al., 2016; Vinha et al., 2016a; López-Hidalgo, 2017), but lacking a validation and effective integration of the different molecular multilevels.

In spite of their difficulty as orphan, recalcitrant plant species, forest trees, like other experimental plant systems, deserve to be considered at the wide system level, that implicates the use of multidisciplinary approaches, from visual phenotype, to molecular – omics, through physiological and biochemical approaches (Correia et al., 2016; Meijón et al., 2016; Escandón et al., 2017). Systems Biology approaches require the optimization of protocols for both wet and *in silico* analysis.

In this direction, trying to fill this gap with the use of the available high-throughput – omics, its combination and also the implementation of required methodology, we hoped to gain knowledge on the chemical composition and metabolism of the *Q. ilex* tree species, its variability among and within populations, the effect on endogenous ones and their environmental factors, and the search for molecular markers to select elite genotypes. The lack of information available in public databases on the Holm oak genome, transcriptome (Guerrero-Sanchez et al., 2017), or proteome (Romero-Rodríguez et al., 2014) and the absence of standardized laboratory and analytical protocols make this approach a real challenge.

In this work, we employed a wide range of *in silico* techniques allowing a system biology approach for a non-sequenced species. To obtain the maximum level of biochemical complexity the plant sample employed were multi-organ pools, generated by mixing equal amounts of homogenized tissue from acorn embryo, leaves, and roots. In setting up protocols for transcriptome (NGS-Illumina), proteome (shotgun LC-MS/MS) and metabolome (GC-MS) analysis, and bioinformatic pipelines for annotating transcripts, proteins and metabolites, the Holm oak metabolic pathways were partially reconstructed. This research constitutes the basis for ongoing and future studies to obtain a better understanding of the molecular bases underlying phenotypes of interest (productive, tolerant to environmental cues, nutraceutical value) and the selection of elite genotypes to be used in restoration and reforestation programs, especially in the current climate change scenario. In order to reveal the particularities of the species under study, data have been compared with those reported for model plant species, including *Arabidopsis*, rice, poplar, and eucalyptus.

## Materials and Methods

### Plant Material

Mature acorns from Holm oak (*Quercus ilex* L. subsp. ballota [Desf.] Samp.) were collected on December 2015 from a tree located in Aldea de Cuenca (province of Córdoba, Andalusia, Spain). Acorns were transported to the lab, sterilized, and germinated as previously reported (Simova-Stoilova et al., 2015). Germinated seeds were sown in pots (500 mL) with perlite and grown in a greenhouse under natural conditions for 4 months up to the 10-leaves stage. Plants were periodically watered at field capacity and once a week with a Hoagland nutrient (Hoagland and Arnon, 1950) solution after the second month. Germinated embryos, cotyledons, leaves, and roots were collected separately, washed with distilled water and frozen in liquid nitrogen. Then, each tissue was separately homogenized in a mortar until a fine powder was obtained and finally stored at -80°C. The experiments were performed with a pool of fresh weight equivalents of the homogenized tissue from acorn embryo, cotyledons, leaves, and roots. Depending on the organ, samples from individual trees or plantlets in number of 18 (roots and leaves) to 50 (seed embryos and cotyledons) were collected and mixed. Three independent extractions were performed and only consistent proteins or metabolites, those present in the three replicates, were considered.

### Transcriptomics Analysis

#### RNA Extraction and Sequencing

Total RNA was extracted from the frozen homogenized pool tissue following the procedure previously reported by (Guerrero-Sanchez et al., 2017). 50 mg pooled fresh tissue according the procedures previously set up in our laboratory for *Q. ilex* samples was employed (Echevarría-Zomeño et al., 2012). Contaminating genomic DNA was removed by DNase I (Ambion) treatment. Total RNA was quantified spectrophotometrically (DU 228800 Spectrophotometer, Beckman Coulter, TrayCell Hellma GmbH & Co., KG. The high quality and integrity of the RNA preparation were tested electrophoretically (Agilent 2100 Bioanalyzer). Only high-quality RNAs with RIN values >8 and A_260_:A_280_ ratios near 2.0 were used for subsequent experiments.

The library construction of cDNA molecules was carried out using Illumina TruSeq Stranded mRNA Library Preparation Kit according to the manufacturer’s instructions using 2 μg of total RNA followed by poly-A mRNA enrichment using streptavidin coated magnetic beads and thermal mRNA fragmentation. The cDNA was synthesized, followed by a chemical fragmentation (DNA library) and sequenced in the Illumina Hiseq 2500 platform, using 100 bp paired-end sequencing (De Wit et al., 2012).

#### Data Processing

The raw reads obtained from the sequencing platform were preprocessed to retain only high-quality sequences to be subsequently used in the assembly. Each original sequence was quality trimmed considering several parameters (quality trimming based on minimum quality scores, ambiguity trimming to trim off, for example, stretches of Ns, base trim to remove specified number of bases at either 3′ or 5′ end of the reads). The processed reads were assembled *de novo* using the assembly software MIRA 4.9.6 (Chevreux and Suhai, 1999). Redundancy reduction of the assembled sequenced was carried out by using the CD-HIT 4.6 clustering algorithm (Li et al., 2001, 2002).

#### Gene Ontology

Assembled sequences were blasted against UniRef90 (UniProt^1^) using the software Sma3s (Casimiro-Soriguer et al., 2017) in order to obtain the annotated sequences with the most probable gene name and protein description, EC numbers for enzymes, GO terms, and UniProt keywords and pathways. In addition, their functions were identified using MERCATOR^2^.

### Proteomics Analysis

#### Protein Extraction and Digestion

Proteins were extracted from the frozen homogenized pool tissue by using the TCA-acetone-phenol protocol as reported in Jorrin-Novo et al. (2014). Protein extracts [600–1000 ng BSA equivalents quantified with Bradford assay (Bradford, 1976)] were subjected to Orbitrap analysis after SDS–PAGE (12%) prefractionation. Electrophoresis was stopped when the sample entered the resolving gel, so that a unique protein band was revealed after Coomassie staining (Pascual et al., 2017).

Protein bands were manually excised, destained, and digested with trypsin Sequencing grade (Roche) as is described in Castillejo et al. (2015) with minor modifications. Briefly, gel plugs were destained by incubation (twice for 30 min) with a solution containing 100 mM ammonium bicarbonate (AmBic)/50% acetonitrile (AcN) at 37°C. Then, they were dehydrated with AcN and incubated in 100 mM AmBic containing first 20 mM DTT for 30 min, and then in the same solution containing 55 mM Iodoacetamide instead DTT for 30 min. They were washed with 25 mM AmBic and 25 mM AmBic/50% AcN two times each. After dehydration in AcN, the trypsin at a concentration of 12.5 ng/μl was added in a buffer containing 25 mM NH_4_HCO_3_, 10% AcN and 5 mM CaCl_2_, and the digestion proceeded at 37 C for 12 h. Digestion was stopped, and peptides were extracted from gel plugs by adding 10 μL of 1% (v/v) trifluoroacetic acid (TFA) and incubating for 15 min.

#### Shotgun LC-MS Analysis

Nano-LC was performed in a Dionex Ultimate 3000 nano UPLC (Thermo Scientific) with a C18 75 μm × 50 Acclaim Pepmam column (Thermo Scientific). The peptide mix was previously loaded on a 300 μm × 5 mm Acclaim Pepmap precolumn (Thermo Scientific) in 2% AcN/0.05% TFA for 5 min at 5 μL/min. Peptide separation was performed at 40°C for all runs. Mobile phase buffer A was composed of water, 0.1% formic acid. Mobile phase B was composed of 80% AcN, 0.1% formic acid. Samples were separated during a 60-min gradient ranging from 96% solvent A to 90% solvent B and a flow rate of 300 nL/min.

Eluted peptides were converted into gas-phase ions by nano electrospray ionization and analyzed on a Thermo Orbitrap Fusion (Q-OT-qIT, Thermo Scientific) mass spectrometer operated in positive mode. Survey scans of peptide precursors from 400 to 1500 *m*/*z* were performed at 120K resolution (at 200 *m*/*z*) with a 4 × 10^5^ ion count target. Tandem MS was performed by isolation at 1.2 Da with the quadrupole, CID fragmentation with normalized collision energy of 35, and rapid scan MS analysis in the ion trap. The AGC ion count target was set to 2 × 10^3^ and the maximum injection time was 300 ms. Only those precursors with charge state 2–5 were sampled for MS^2^. The dynamic exclusion duration was set to 15 s with a 10 ppm tolerance around the selected precursor and its isotopes. Monoisotopic precursor selection was turned on. The instrument was run in top 30 mode with 3 s cycles, meaning that the instrument would continuously perform MS^2^ events until a maximum of top 30 non-excluded precursors or 3 s, whichever was shorter.

#### Protein Identification

Spectra were processed using the SEQUEST algorithm available in Proteome Discoverer© 1.4 (Thermo Scientific, United States). The following settings (Romero-Rodríguez et al., 2014) were used: precursor mass tolerance was set to 10 ppm and fragment ion mass tolerance to 0.8 Da. Only charge states + 2 or greater were used. Identification confidence was set to a 5% FDR and the variable modifications were set to: oxidation of methionine and the fixed modifications were set to carbamidomethyl cysteine formation. A maximum of two missed cleavages were set for all searches. The protein identification, was carried out against the annotated *Q. ilex* transcriptome, previously described. A six-frame translation for each sequence in the transcriptome was performed by using EMBOSS (Rice et al., 2000), filtering and keeping peptides longer than 50 amino acids. Considering the identified proteins, the protein peak areas were normalized and missing values corrected. Mean values and standard deviation (SD), as well as the coefficient of variation (CV) of the peak areas of protein species were determined for three independent analysis (**Supplementary Table S8**). The remaining sequences were used as a database for the protein identifications and their functions were identified using MERCATOR (Lohse et al., 2014).

### Metabolomics Analysis

#### Metabolite Extraction

Metabolites were extracted from plant tissue as described by Valledor et al. (2014), with three independent extractions. A buffer containing 600 μL of cold methanol: chloroform: water (5:2:2) was added to 15 mg of frozen tissue, vortexed (10 s), and the mixture sonicated (ultrasonic bath, 40 kHZ for 10 min). After centrifugation (4°C, 4 min, 20,000 × *g*) the supernatant was transferred to new tubes containing 400 μL of cold chloroform: water (1:1). For phase separation, the tubes were centrifuged (4°C, 4 min, 20,000 × *g*). The upper (polar) and the lower (apolar) phases were re-extracted with 200 μL of cold chloroform (upper) and water (lower), respectively. After combining on one hand the water: methanol (upper) and, on the other the chloroform (lower) phases, they were vacuum dried at 25°C (Speedvac, Eppendorf Vacuum Concentrator Plus/5301).

#### GC-MS Analysis

GC-MS analysis was performed as reported Furuhashi et al. (2012) and Meijón et al. (2016) with some modifications. Polar (water: methanol dissolved) metabolites were derivatized by re-suspending the dried extract in 20 μL of anhydrous pyridine containing 40 mg/mL of methoxyamine hydrochloride. The mixture was incubated at 30°C for 30 min under agitation. Next, 60 μL of *N*-methyl-*N*-trimethylsilyl trifluoroacetamide (MSTFA) was added, samples incubated at 60°C for 30 min, centrifuged (3 min, 20,000 × *g*), and cooled to room temperature. Then, 80 μL of the supernatant was transferred to GC-microvials. Apolar (chloroform solubilized) metabolites were methylesterified with 295 μL tert-methyl-Butyl-Ether (MTBE), and 5 μL of trimethylsulfonium hydroxide solution (TMSH) for 30 min at room temperature. The tubes were centrifuged (3 min, 20,000 × *g*) to remove insoluble particles before transferring the supernatants to GC-microvials.

Polar metabolites were resolved and analyzed with a Gas Chromatograph/Mass Spectrometer Agilent 5975B GC/MSD. Inlet temperature was set at 230°C. Samples were injected in discrete randomized blocks with a 1.2 mL/min flow rate. GC separation was performed splitless on a HP-5MS capillary column (30 m × 0.25 mm × 0.25 mm) (Agilent 19091J-433) over a 70–76°C gradient at 0.75°C/min, 76–180°C gradient at 6°C/min, 180–200°C gradient at 3.5°C/min, and then to 310°C at 6°C/min. The mass spectrometer operated in electron-impact (EI) mode at 70 eV in a scan range of *m*/*z* 40–800. For apolar metabolites a different temperature gradient was employed: 80–190°C at 8°C/min, 190–220°C at 5°C/min, and then to 270°C at 5°C/min. The mass spectrometer was operated in EI mode at 70 eV in a scan range of *m*/*z* 40–600.

#### Metabolite Identification

Metabolites were “tentatively assigned” based on GC retention times (RT) and *m*/*z* values (**Supplementary Tables S1, S2**) through searches in different databases, including the Gölm Metabolome Database (Nielsen and Jewett, 2007), Alkane, Fiehn library 1 y 2 (Kind et al., 2010), GC-TSQ, MoSys, and *NIST*/EPA/NIH Mass Spectral Library. Three different softwares were used for metabolite identification: MZmine 2 (2.24 version^3^) (Pluskal et al., 2010), AMDIS software (2.66 version^4^), and NIST.MS Search (2.01 version^5^). Mean values and SD, as well as the CV of the peak areas of metabolites were determined for three independent extraction (**Supplementary Table S2**). Moreover, the metabolites were annotated using the KEGG compound reference database^6^. Metabolomics pathways of each metabolite (**Supplementary Table S3**) were searched against KEGG pathway maps^7^. For other general biological networks, we employed MapMan (3.5.1 version^8^).

### Interspecies Comparison

The annotated *Q. ilex* transcriptome was compared against the complete *in silico* proteomes of *Arabidopsis thaliana* (UP000006548^9^, *Oryza sativa subsp. japonica* (UP000059680^10^), *Populus trichocarpa* (UP000006729^11^), and *Eucaliptus grandis* (UP000030711^12^) in order to elucidate the unique and shared sequences. This comparison was performed by using BLAST^13^ with blastX alignment with an *e*-value of 10^-10^. Also, the EC numbers of each proteome were contrasted to achieve a complete picture of the metabolic pathways coverage differences among proteomes studied in previously mentioned species (**Supplementary Table S4**). For the comparison, we represented a Venn diagram plotted using VennDiagram R package (Chen and Boutros, 2011).

### Integrated Pathway

By using MERCATOR web application^14^ (**Supplementary Tables S5, S6**) (Lohse et al., 2014), we could assign MapMan “Bins” to arbitrary transcript or protein input sequences (Usadel et al., 2009). The output was a text file mapping each input (proteins or transcripts) identifier to one or more Bins by searching a variety of reference databases (TAIR Release 10, SwissProt/UniProt Plant Proteins, Clusters of Orthologous Eukaryotic Genes Database (KOG), Conserved Domain Database (CDD), and InterProScan). The functional predictions generated could directly be used as a “mapping file” for the high-throughput data visualization and meta-analysis software MapMan (3.5.1 version^15^. The ImageAnnotator module allowed us to visualize the data on a gene-by-gene basis on schematic diagrams (maps) of the biological processes described.

## Results and Discussion

This paper reports the study and view of the metabolism as it occurs in Holm oak, the most representative and valuable forest tree species in the Mediterranean region. For that purpose, a biological sample containing equal fresh weight amount of the different organs as starting plant material and a combination of high-throughput, -omics approaches (transcriptomics, proteomics, and metabolomics) as analytical tools were used. As each analytical platform has its own limitations (Schrimpe-Rutledge et al., 2016; Tian et al., 2016; Viant et al., 2017), is their integration that will provided more confident biological knowledge of them.

The Systems Biology approach for research with species that, like Holm oak are orphan and recalcitrant is very challenging (Abril et al., 2011), and it required the optimization of experimental protocols and, more limitative, the creation of custom-made databases, and pipelines. Beyond the reconstruction of different metabolic pathways as they may occur in Holm oak, and the comparison with model plant species (*A. thaliana, O. sativa subsp. japonica, P. trichocarpa*, and *E. grandis*) we aimed to prove that employing state-of-the-art instrumentation and a similar workflow to those employed in model species is feasible, even though quite uncommon in the current literature.

### Transcriptome Analysis

**FIGURE 1:**
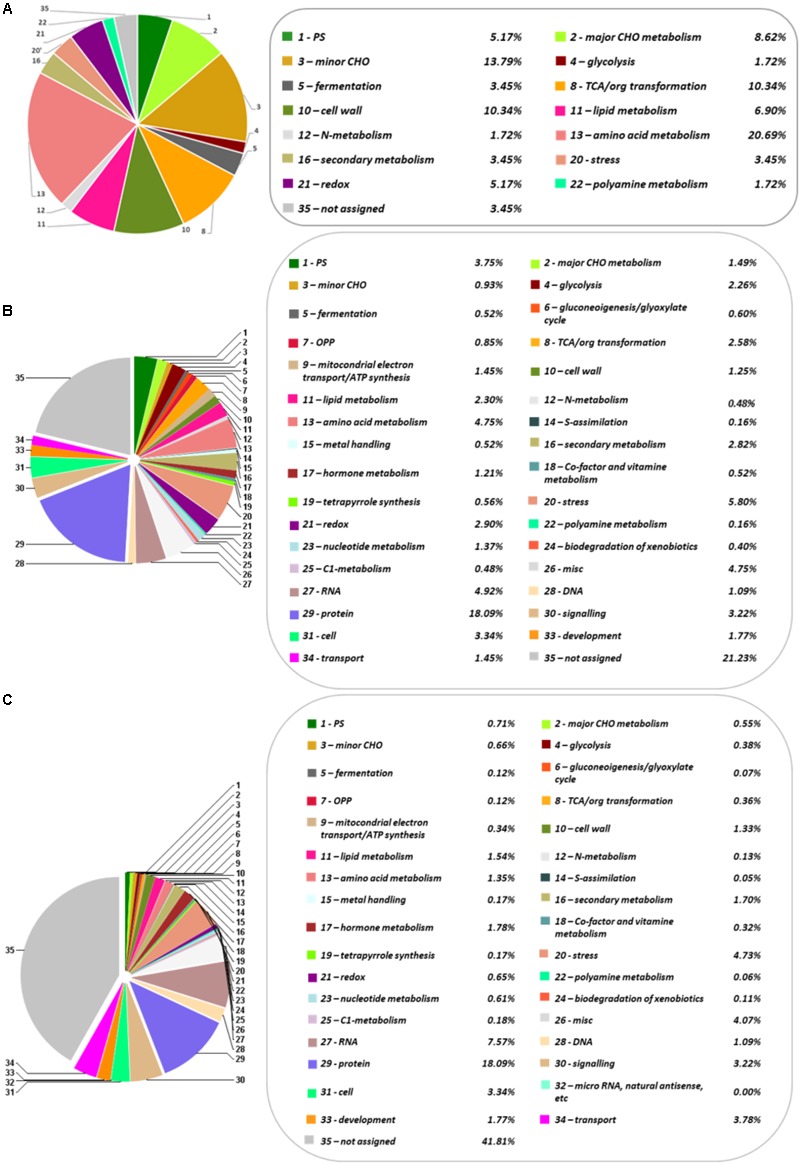
Functional categorization and distribution in percentage of the identified metabolites, proteins and transcripts, according to the categories establish by MERCATOR. **(A)** Metabolome. **(B)** Transcriptome. **(C)** Proteome. The pie charts show different functional categories: PS (*Photosynthesis*), major CHO metabolism, minor CHO metabolism, glycolysis, fermentation, gluconeogenesis/glyoxylate cycle, OPP (*Oxidative Pentose Phosphate*), TCA/org transformation, mitochondrial electron transport/ATP synthesis, cell wall, lipid metabolism, N-metabolism, amino acid metabolism, S-assimilation, metal handling, secondary metabolism, hormone metabolism, co-factor and vitamin metabolism, tetrapyrrole synthesis, stress, redox, polyamine metabolism, nucleotide metabolism, biodegradation of xenobiotics, C1-metabolism, miscellanea, RNA, DNA, protein, signaling, cell, micro RNA, natural antisense, etc., development, transport, and not assigned.

The first transcriptome of *Q. ilex* has recently been reported. For that reason, the Illumina Hiseq 2500 platform was employed to analyze the tissue mix sample, resulting in 119889 contigs, and 31973 Blast2GO annotated transcripts (Guerrero-Sanchez et al., 2017). The number of annotated sequences have been increased to 62628 after a UniRef90 database search through Sma3s software (Muñoz-Mérida et al., 2014; Casimiro-Soriguer et al., 2017). Among them, 27089 sequences corresponded to unique genes. Comparatively, Sma3s performed faster than Blast2GO and allowed more elaborated results, including functional categories, such as biological processes, cellular components or molecular functions (**Supplementary Figures S1–S3**). The total transcriptome sequences were categorized in 35 MERCATOR functional plant categories. The result of this categorization showed a high percentage (41.8%) of non-assigned transcripts (**Figure 1**). Response to stress and biosynthetic process, and the nucleus and plastids, were, respectively, the biological processes and organelles most represented (**Supplementary Figures S1, S2**). With respect to molecular functions, ion binding and kinase activity were those most abundant, with around 11225 and 6372 sequences, respectively (**Supplementary Figure S3**).

The number of annotated transcripts, 62628, is double that previously found for the close relative *Q. robur* (38292 sequences; Tarkka et al., 2013), similar to the figure of 27655 protein-coding genes in *Arabidopsis* (35386 identified proteins; Araport11^16^), and below the 82190 unique transcripts corresponding to 34212 genes also reported in *Arabidopsis* by Zhang et al. (2017).

**FIGURE 2:**
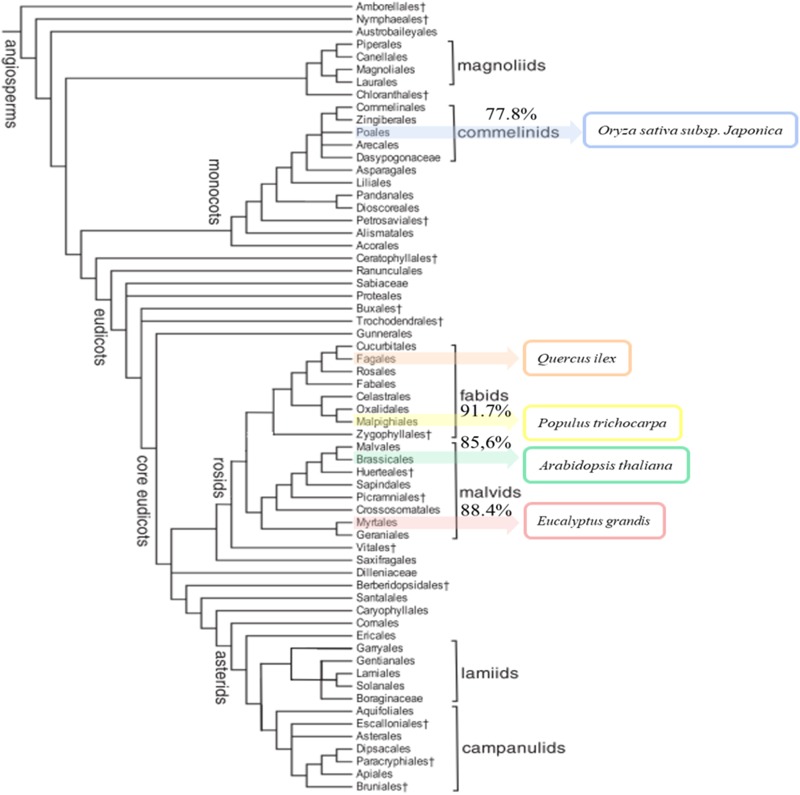
Phylogenetic tree of angiosperms. The tree shows the five-species compared (*Arabidopsis thaliana, Eucalyptus grandis, Oryza sativa subsp. japoni*ca, *Populus trichocarpa*, and *Quercus ilex*). The sequence similarity of species coincides with the classification in the phylogenetic tree. Species are ranked from highest to lowest similar to *Q. ilex: P. trichocarpa* (91.7%), *E. grandis* (88.4%), *A. thaliana* (85.6%), and *O. sativa subsp. japoni*ca (77.8%).

The annotated sequences in *Q. ilex* transcriptome were compared with the *in silico* proteomes *of A. thaliana, O. sativa subsp. japonica, P. trichocarpa*, and *E. grandis* (UniProt) to elucidate the unique and shared sequences. The comparative results are shown in **Supplementary Table S2**. The highest percentage of similarity corresponded to *P. trichocarpa* (91.7%), and lowest to *O. sativa subsp. japonica* (77.8%), with intermediate values for *E. grandis* (88.5%) and *A. thaliana* (85.6%). The percentage of similarity correlated with the phylogenetic distances among the compared species as reported by The Angiosperm Phylogeny Group III (2009) (**Figure 2**).

**FIGURE 3:**
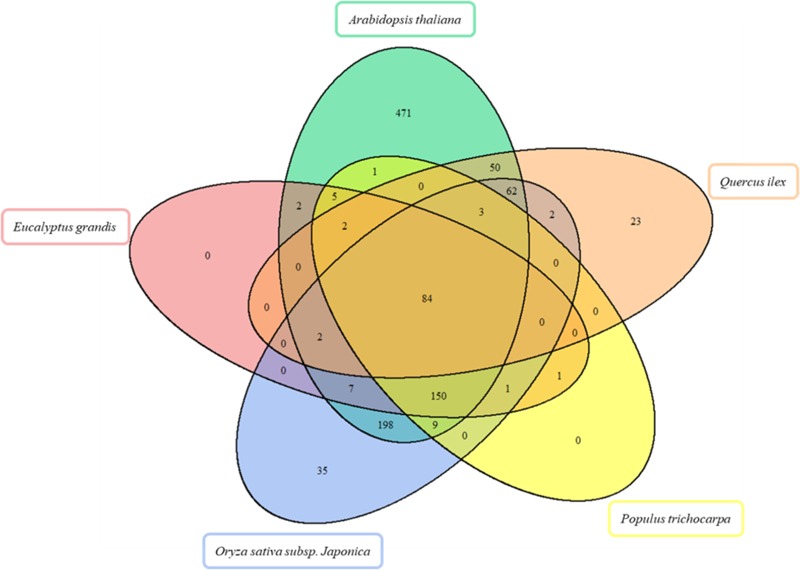
Venn diagram for the comparison of enzymes in *Arabidopsis thaliana, Eucalyptus grandis, Oryza sativa subsp. japoni*ca, *Populus trichocarpa in silico* proteomes, and *Quercus il*ex proteome. The Venn diagram shows the overlap of enzymes detected.

Among the annotated transcripts, 2103 corresponded to enzyme transcript products. These enzymes were assigned to 123 KEGG metabolic pathways (**Supplementary Table S3**). The most represented pathways (**Table 2**) were: the carbohydrate metabolism (starch and sucrose metabolism and glycolysis/gluconeogenesis, with 26 and 30 enzyme transcripts, respectively). Also, the amino acids metabolism, primarily the cysteine and methionine metabolism, where 37 enzyme transcripts were detected. This pathway has an important role in plants. Cysteine constitutes the sulfur donor for the biosynthesis of methionine, phytochelatins, sulfhydryl compounds, glutathione, and coenzymes. The homeostasis of sulfur metabolism in trees is more robust than in herbaceous plants. Also, a greater change in conditions to initiate a response in trees is required (Rennenberg et al., 2007). This fact is coherent with the requirement for highly flexible defense strategies in woody plant species because of longevity. In addition, the lipid metabolism (glycerophospholipid metabolism with 32 enzyme transcripts) has an important function as a mediator in hormone signal transduction in plants (Janda et al., 2013).

### Proteome Analysis

The protein profile of the *Q. ilex* tissue mix sample was analyzed using a shotgun proteomics platform. Protein extracts were obtained by using a TCA-acetone-phenol protocol. After trypsin digestion, peptides were subjected to UPLC-Q-OT-qIT MS. The resulting peptides and corresponding proteins were identified by matching MS and MS/MS *m*/*z* data against the protein database resulting from the six-frame translation of the *Q. ilex* transcriptome. The employment of species specific databases instead of generic Viridiplantae ones improved the number and confidence of the identifications, as previously published (Romero-Rodríguez et al., 2014). By using Viridiplantae (SwissProt), 891 proteins were identified. Nevertheless, with our custom-built specific database, 58584 peptides were detected corresponding to 2830 proteins (with at least one unique peptide (**Supplementary Tables S7, S8**). Mean, SD, and CV (%) values of normalized identified protein peak areas were determined for three replicates (**Supplementary Table S8**). The mean of the CV obtained was 36.75% (**Supplementary Table S8**), which was slightly higher than the CV mean previously described using a 2-DE gel analysis (28.9%) (Jorge et al., 2005, 2006). This is due to the number proteins, considering that this number is much lower in a 2-DE gel analysis and usually highly represented than in a shotgun LC-MS/MS. However, despite having a slightly higher value of CV, the shotgun LC-MS/MS shows greater sensitivity and wide dynamic range. Proteins were categorized in 34 MERCATOR functional plant categories (**Figure 1**). 21.2% of the proteins was not assigned to a functional plant category. Up-to 18.1% proteins were related to protein fate (assembly, folding, degradation, and protein posttranslational modifications), this group being the one most represented.

The Holm oak proteome was filtered manually looking for proteins corresponding to enzymes based on the EC number. This resulted in 228 enzyme proteins, corresponding to 10% of the protein species with EC deduced from the *in silico* predicted Holm oak transcriptome (2103 enzyme proteins) and around 20–50% of the enzymes predicted for the sequenced *A. thaliana* and *O. sativa subsp. japonica* systems at UniProt.

The proteins identified were assigned to 93 KEGG metabolic pathways (**Supplementary Table S3**). The most represented pathways were: the carbohydrate metabolism (starch and sucrose metabolism and glycolysis/gluconeogenesis) and the amino acids metabolism (**Table 2**). The least represented one was the enzymes related to transcription (**Supplementary Table S3**). These figures are much higher than those previously reported for *Q. ilex* and other forest tree species (Valero-Galván et al., 2012b; Pascual et al., 2017; Szuba and Lorenc-Pluciñska, 2017), maybe due to the use of the powerful LTQ-Orbitrap mass instrument (Kalli et al., 2013) and the search in custom-built specific database.

Out of the 228 enzyme proteins identified, 23 were specific for Holm oak, and 202, 157, 88, and 87, shared with, respectively, *A. thaliana, O. sativa subsp. japonica, P. trichocarpa*, and *E. grandis* (**Figure 2**). 84 enzymes were common to all the species, and 471, and 35 specific for *A. thaliana* and *O. sativa subsp. japonica*. It is worthnoting that, for *P. trichocarpa* and *E. grandis* no unique enzymes were found, this proving the quality and validity of our data, with, consequently, a more complete annotated transcriptome and proteome database. Holm oak unique enzymes were related to the biosynthesis of hormones and secondary metabolites. They included those involved in the zeatin biosynthetic pathway (*ath00908*), such as *cis*-zeatin *O*-beta-D-glucosyltransferase (EC:2.4.1.215) and zeatin *O*-beta-D-xylosyltransferase (EC:2.4.2.40). Zeatin, one of the growth promoting hormones, is the predominant xylem-mobile cytokinin in many plant species (Kamboj et al., 1999). In the Holm oak unique enzymes involved in the secondary metabolism [6′-deoxychalcone synthase (EC:2.3.1.170) and prenylcysteine oxidase (EC:1.8.3.5)] were involved in flavonoid biosynthesis and terpenoid backbone biosynthesis, respectively. This is not surprising as secondary metabolites are species specific. Thus, in Holm oak, the flavonoids epicatechin gallate and epigallocatechin were found (Vinha et al., 2016b).

The 84 enzyme proteins common to the five-species corresponded mostly to pathways of the central metabolism, such as those of starch and sucrose (e.g., sucrose synthase, EC: 2.4.1.13, and glucose-6-phosphate isomerase, EC: 5.3.1.9), glycolysis and gluconeogenesis [e.g., phosphoglycerate kinase (EC:2.7.2.3) and pyruvate kinase. (EC:2.7.1.40)], and citrate cycle [e.g., malate dehydrogenase (EC:1.1.1.37), pyruvate dehydrogenase (EC:1.2.4.1), and aconitate hydratase (EC:4.2.1.3)].

The 228 enzyme proteins identified belonged to 109 pathways, with some of them being represented by only one enzyme [e.g., caffeine metabolism (*ath00232*) and arachidonic acid metabolism (*ath00590*)] and up to 20 enzymes [e.g., carbon fixation in photosynthetic organisms (*ath00710*)]. Analysis of the UniProt in silico enzyme proteome revealed 106 and 107 pathways for, respectively, *P. trichocarpa* and *E. grandis*, with the figure being higher for *A*. *thaliana* (121 pathways) and *O. sativa subsp. japonica* (112 pathways) (**Supplementary Table S8**).

The pathways most represented in Holm oak were those of the intermediate and central metabolism, including glyoxylate and dicarboxylate metabolism (*ath00630*) with 16 enzyme proteins and amino sugar and nucleotide sugar metabolism (ath00520) with 12 enzyme proteins (**Table 2**). For the glycolysis (**Supplementary Figure S4**), just as an example, there were only two enzyme proteins non-detected: phosphofructokinase (EC:2.7.1.11) and phosphoglycerate mutase (EC:5.4.2.12) (**Supplementary Table S9**). These results are more complete than the ones found from the *in silico* analysis of the other two woody plants used for comparisons *P. trichocarpa* and *E. grandis*, with only 5 out of the 10 glycolytic enzymes.

### Metabolome Analysis

The metabolites present in the pooled samples were analyzed by using GC-q-MS. Two different extraction solvents, methanol:water and chloroform, were, respectively, used for compounds of different polarities. Up to 155 and 19 peaks were resolved by gas chromatography using the above mentioned solvents. A complete list of the identified compounds with their respective RT and the mass-to-charge ratios (*m*/*z*) is included in **Supplementary Tables S1, S2**. From the m/z values, and after a search in seven public databases (Alkane, Fiehn library 1 and 2, Gölm Metabolome Database, GC-TSQ, MoSys, and NIST/EPA/NIH Mass Spectral Library) a total of 62 compounds were identified, 57 in the methanol:water extract and 5 in the chloroform one. The normalized peak areas of the metabolites were employed for the mean, SD, and CV determinations. The average of the CV obtained (13.70%) was lower than the obtained with proteins data (36.75%), revealing the existence of a greater variability in proteins analysis. The higher CV could be related with the higher number and diversity of identified proteins versus the metabolites identified.

Identified compounds were in the 60–500 Da and mostly belonged to the primary metabolism (59), with only three being secondary metabolites (catechin, epigallocatechin, and anthraquinone). The identified metabolites were grouped in six chemical families according to the KEGG database^17^, including carbohydrates (19), organic acids (19), amino acids (11), fatty acids (4), polyols (2), and phenolic compounds (2) (**Table 1**). The family most represented was that of organic acids (19) and carbohydrates (19), followed by amino acids (11). Fatty acids (4) and phenolic compounds (2) were much less represented. They were included in at least 64 different KEGG pathways (**Supplementary Table S3**), and in 15 functional plant categories according to MapMan classification (**Figure 1**).

**Table 1:** Metabolite families from GC-MS data of *Quercus ilex*.

Nature of the compounds	Metabolite name
Amino acids	L-Glutamate (C00025), L-aspartate (C00049), L-alanine (C00041), L-asparagine (C00152), L-serine (C00065), L-threonine (C00188), L-leucine (C00123), L-valine (C00183), L-isoleucine (C00407), L-proline (C00148), L-phenylalanine (C00079)
Organic acids	Ascorbate (C00072), pyruvate (C00022), L-lactate (C00186), succinate (C00042), fumarate (C00122), malate (C00149), citrate (C00158), aconitate (C00417), gluconolactone (C00198), D-glycerate (C00258), glucarate (C00818), galactarate (C00879), maleate (C01384), salicylate (C00805), pyroglutamic acid (C01879), oxalate (C00209), gallate (C00627), quinate (C00296), D-ribonate (C01685)
Carbohydrates	D-Glucose (C00031), L-arabinose (C00259), D-xylulose (C00310), D-galacturonate (C00333), D-fructose (C00095), L-sorbose (C00247), mannitol (C00392), L-rhamnose (C00507), D-sorbitol (C00794), sucrose (C00089), D-galactose (C00124), melibiose (C05402), myo-inositol (C00137), D-glucose 6-phosphate (C00092), maltose (C00208), maltotriose (C01835), D-cellobiose (C00185), D-galactonate (C00880), D-erythrose (C01796)
Polyols	Glycerol (C00116), viburnitol (C08259)
Fatty acids	Palmitic acid (C00249), oleic acid (C00712), stearic acid (C01530), linoleic acid (C01595)
Phenolic compounds (flavonoids)	Catechin (C06562), epigallocatechin (C12136)
Others	Urea (C00086), 4-aminobutanoate (GABA) (C00334), tridecane (C13834), anthraquinone (C16207)

*Six main chemical families of metabolites are represented. Carbohydrates (19), organic acids (19), amino acids (11), fatty acids (4), polyols (2), phenolic compounds (2), and four unique compound classes (others). Data in the brackets are KEGG compound identifier of each metabolite.*

**FIGURE 4:**
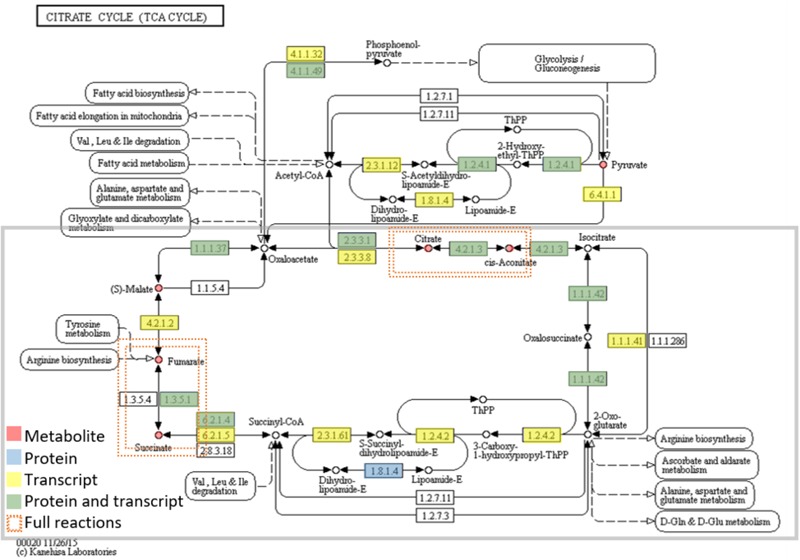
Metabolites and enzymes (protein or transcript level) assigned to the citrate cycle (TCA cycle). Omics data are highlighted in red (metabolites), blue (proteins), yellow (transcripts), and green (both proteins and transcript). The enzymes (proteins and transcripts) are named by their EC number. EC numbers and respective detected TCA cycle enzymes: 2.3.3.1 (*Citrate synthase*), 4.2.1.3 (*Aconitate hydratase*), 1.1.1.42 [*Isocitrate dehydrogenase (NADP^+^)*], 1.2.4.2 (*alpha-ketoglutarate dehydrogenase*), 6.2.1.4 (*Succinyl coenzyme A synthetase*), 1.3.5.1 (*Succinate dehydrogenase*), 4.2.1.2 (*Fumarate hydratase*), 1.1.1.37 (*Malate dehydrogenase*). There are two full reactions (metabolite, protein and transcript level) This figure was adapted from KEGG reference pathway.

These metabolites are starting metabolites or final products from primary metabolism pathways, like glyoxylate and dicarboxylate metabolism (*ath00630*), starch and sucrose metabolism (*ath00500*), citrate cycle (TCA cycle) (*ath00020*) of carbohydrate metabolism; alanine, aspartate, and glutamate metabolism (*ath00250*) of amino acid metabolism and biosynthesis of unsaturated fatty acids (*ath01040*) of fatty acids metabolism. Many were intermediate metabolites, with 5 (citrate, *cis*-Aconitate, succinate, fumarate, and malate), out of the total 8 corresponding to the Citrate cycle (**Figure 4** and **Table 2**). The pathways most represented were carbohydrate and amino acid metabolisms. However, the number of secondary metabolites (catechin, epigallocatechin, and anthraquinone) was smaller than the number of secondary metabolites reported for *Quercus* spp. acorns (Vinha et al., 2016a). Due to the small number of secondary metabolites detected, the metabolic pathways related to the biosynthesis of secondary metabolites, like carotenoid biosynthesis (*ath00906*), anthocyanin biosynthesis (*ath00942*), and monoterpenoid biosynthesis (*ath00902*) are not highly represented (**Supplementary Table S3**). In *Arabidopsis*, the total number of secondary metabolites is still unknown due to metabolite identification being one of the bottlenecks in untargeted metabolomic studies (Wu et al., 2017). Still, in AraCyc 15.0, the total number of compounds described are 2971 and the number of metabolic pathways 610 (PMN; Plant Metabolic Network^18^).

**Table 2:** Number of metabolites and enzymes (proteomic and transcriptomic level) in KEGG pathways.

Pathways	Metabolites		Proteins	Transcripts
Carbohydrate metabolism		
Glycolysis/gluconeogenesis (*ath00010*)	Pyruvate, D-glucose, L-lactate	3	20	30
Glyoxylate and dicarboxylate metabolism (*ath00630*)	Pyruvate, L-glutamate, succinate, L-serine, malate, citrate, glycolate, oxalate, glycerate, aconitate	10	16	27
Citrate cycle (TCA cycle) (*ath00020*)	Pyruvate, succinate, fumarate, malate, citrate, aconitate	6	9	16
Amino sugar and nucleotide sugar metabolism (*ath00520*)	D-Glucose, L-arabinose, D-galacturonate	3	12	38
Starch and sucrose metabolism (*ath00500*)	D-Glucose, sucrose, D-glucose 6-phosphate, D-fructose, cellobiose, maltose	6	18	26
Pentose phosphate pathway (*ath00030*)	Pyruvate, D-glucose, gluconolactone, glycerate	4	7	17
Galactose metabolism (*ath00052*)	D-Glucose, sucrose, D-fructose, glycerol, D-galactose, myo-inositol, D-sorbitol, D-galactonate, melibiose	9	9	15
Amino acid metabolism		
Alanine, aspartate, and glutamate metabolism (*ath00250*)	Pyruvate, L-glutamate, L-alanine, succinate, L-aspartate, fumarate, L-asparagine, citrate, 4-aminobutanoate (GABA)	9	9	27
Cysteine and methionine metabolism (*ath00270*)	Pyruvate, L-alanine, L-aspartate, L-serine	4	10	37
Glycine, serine, and threonine metabolism (*ath00260*)	Pyruvate, L-aspartate, L-serine, L-threonine, glycerate	5	11	31
Phenylalanine metabolism (*ath00360*)	Pyruvate, succinate, L-phenylalanine, fumarate, salicylate	5	4	14
Lipid metabolism		
Biosynthesis of unsaturated fatty acids (*ath01040*)	Palmitic acid, oleic acid, stearic acid, linoleic acid	4	3	13
Energy metabolism		
Carbon fixation in photosynthetic organisms (*ath00710*)	Pyruvate, L-alanine, L-aspartate, malate	4	20	23
Biosynthesis of other secondary metabolites		
Phenylpropanoid biosynthesis (*ath00940*)	L-Phenylalanine, gallate	2	7	17

*Pathways according to the KEGG pathway maps based on Arabidopsis thaliana. The Arabidopsis pathway identifiers are in brackets. The table shows the most representative pathways. The complete list of pathways is in the **Supplementary Material Table S9**.*

The identification of 62 metabolites is in the order of what has been reported for non-model plant systems by using a similar approach (Warren et al., 2012; Cadahía et al., 2014, Asai et al., 2016; Pascual et al., 2017), but far from the figure obtained when using model systems such as *A. thaliana*, or complementary techniques such as LC-MS. The employment of complementary LC-MS strategies would increase the number of metabolites identified, as shown, for example, with *A. thaliana*, although it would greatly reduce the number of metabolites identified with no doubts. Kim et al. (2015) detected 4483 distinct metabolite peaks from leaves using 11 mass spectrometric platforms, but only identifying 1348 metabolites. These results revealed that the available databases and repositories are incomplete and pointed to the need for new algorithms for elucidating structures from MS^n^ analyses.

### Data Integration

**FIGURE 5:**
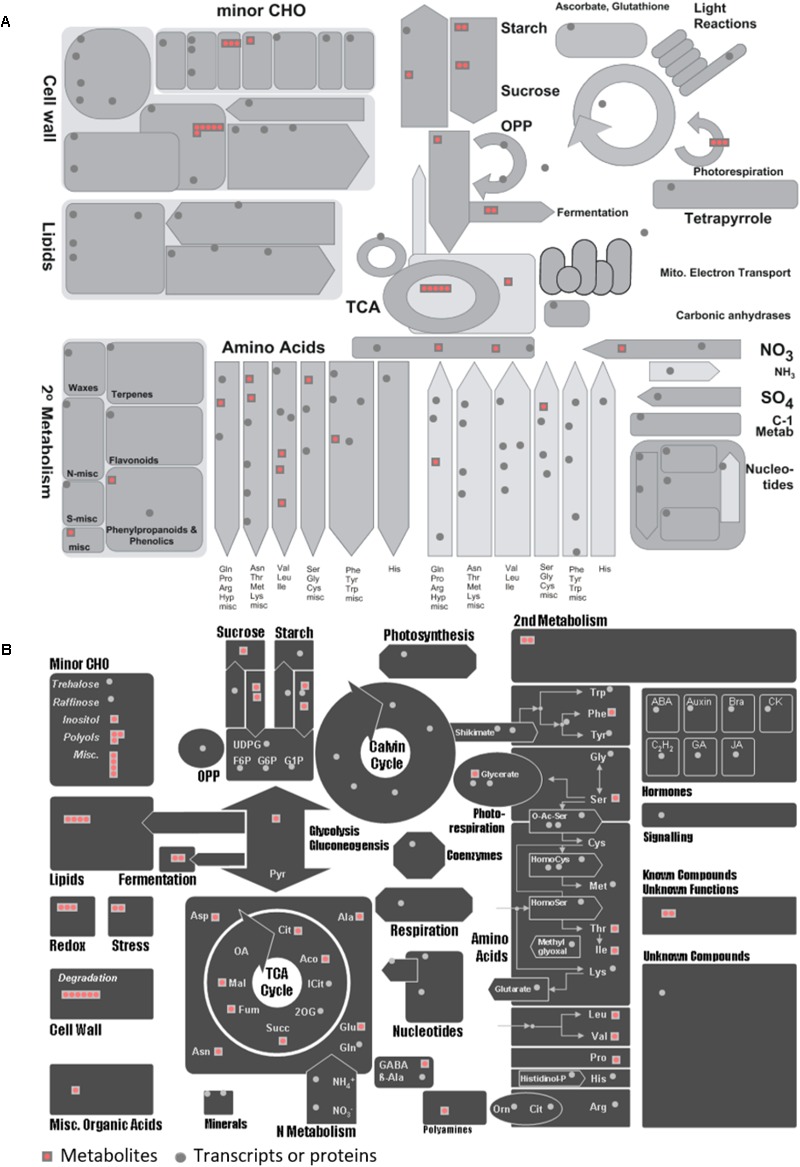
MapMan overview of general metabolism for the metabolites and proteins/transcripts of *Quercus ilex*. **(A)** Visualization of 58 metabolites in the context of general metabolism using the using MapMan software. **(B)** Visualization of 58 metabolites in different MapMan pathways. Each red square represents a metabolite and each gray circle represents a protein or transcript. More details can be found in Usadel et al. (2009).

To seek insights into the metabolic pathways as they occur in Holm oak, transcriptomics, proteomics, and metabolomics data have been integrated. (**Table 1** and **Supplementary Tables S8, S10**). We obtained a deeper view of the metabolic pathways by implementing proteomics or transcriptomics data as the potential of these techniques is much higher than that of metabolomics. However, although technological advances and bioinformatic tools and resources for making those analyses and data interpretation have been extended to plant biology research, this has mostly been for model plants. The unique and specialized biology of such diversified species requires the adaptation of strategies conceived primarily for model organisms and the development of designed and specific methods. For their integration, we employed EC numbers (proteins and transcripts) and KEGG identifiers (metabolites). With the latter and with KEGG pathway maps we obtained the three-different level of information of 61 metabolic pathways (**Supplementary Table S3**). The metabolic pathways most represented are shown in **Table 2**. In order to obtain a metabolic overview. The “BINS” generated from the proteome/transcriptome were employed as a “mapping file,” then introducing identified metabolites. The representation obtained of the general map (**Figure 5A**) for the dataset as shown from ImageAnnotator module of MapMan, showed common metabolism points between metabolites and proteins/transcripts (**Figure 5B**). From the total number of pathways reported in the plants, for example, in KEGG (127 pathways in *Arabidopsis*), we procured data from 124 of them at the metabolomic, proteomic, and transcriptomics level (**Supplementary Figure S3**). **Table 2** summarizes the most representative pathways visualized, including carbohydrate metabolism [glycolysis/gluconeogenesis (*ath00010*), glyoxylate and dicarboxylate metabolism (*ath00630*), citrate cycle (TCA cycle) (*ath00020*), starch and sucrose metabolism (*ath00500*)], amino acid metabolism [alanine, aspartate, and glutamate metabolism (*ath00250*) and phenylalanine metabolism (*ath00360*), lipid metabolism (biosynthesis of unsaturated fatty acids (*ath01040*)], and energy metabolism [carbon fixation in photosynthetic organisms (*ath00710*)]. The one most represented was the TCA, with 5 metabolites out of a total of 10, and protein and transcript corresponding to, respectively, 6 and 8 enzymes (**Figure 2**). On the other hand, there were clear gaps in the hypothetical plant metabolic chart, mainly corresponding to the secondary metabolism and hormones [anthocyanin biosynthesis (*ath00942*), brassinosteroid biosynthesis (*ath00905*)] and lipid metabolism [steroid biosynthesis (*ath00100*)]. For example, the brassinosteroid biosynthesis pathway, which produces plant steroidal hormones that play important roles in many stages of plant growth, has only reported 1 protein and 1 transcript (**Supplementary Table S4**). Also, the **Figure 3** shows the low representation of the different metabolic pathways, also with a multi-omics data integration. From metabolomics, proteomics, and transcriptomic data we were able to identify 64, 109, and 118, pathways, respectively. The total number reported at the PMN and deduced from genome sequencing were 610 (*A. thaliana*), 519 (*E. grandis*), and 538 (*P. trichocarpa*). From these figures we can conclude that the current wet methodologies only allow the visualization of a low percentage of enzyme gene products in a single experiment.

The work and dataset generated, even considering future methodological improvements, will be the basis of ulterior studies on the particularities of the metabolism as it occurs in different organs and developmental processes, as well the changes in response to environmental cues, thus complementing our previous studies in which morphology, phenology, classical physiological and biochemical analysis, and the holistic proteomics have been employed (Echevarría-Zomeño et al., 2009, 2012; Valero-Galván et al., 2011, 2012a; Sghaier-Hammami et al., 2013; Romero-Rodríguez et al., 2014, 2015; Romero-Rodríguez, 2015; Simova-Stoilova et al., 2015; Guerrero-Sanchez et al., 2017). These previously published studies provided quite fragmented and speculative biological information. Hence to go one step ahead, data validation and integration at the different molecular levels would be necessary in order to obtain an unbiased molecular interpretation of the plant biology.

## Conclusion

We have proven that – omics integration, in the Systems Biology direction, is feasible not only with model organisms, but also with orphan and recalcitrant species such as the Holm oak, the most emblematic and representative tree species of the Mediterranean forest. The methodological bases, including wet protocols and *in silico* analysis, have been established, allowing the implementation of transcriptome, proteome, and metabolome databases, comprising 27089 transcripts (unigenes), 2380 protein species, and 62 metabolites (**Supplementary Table S11**).

Integrated analysis allowed the visualization and reconstruction of the metabolism in Holm oak. Up to 123 metabolic pathways, out of the 127-total reported in KEGG, can be visualized at the transcriptome, proteome, and metabolome level. Thus, as an example, for the Krebs cycle, six metabolites out of the eight have been detected. This route comprises eight enzymes detected at the transcriptome or proteome level. These figures are like those reported for the model plant *A. thaliana*. There is still room for improvement, and there are pathways underrepresented in the created database, including the brassinosteroid biosynthesis pathway. The *Q. ilex* genome sequencing, the use of alternative and complementary strategies such as LC-MS will improve the number of pathways visualized.

The current metabolic reconstruction achieved for this species can be considered to be sufficient to progress in the biological knowledge of this species.

## Data Availability

RAW and MSF files corresponding to proteomics are available at the ProteomExchange repository; Datasets: PXD008001. The Project ID of the GC-MS *Q. ilex* metabolomic analysis is PR000618 in the Metabolomics Workbench repository.

## Author Contributions

CL-H performed the GC/MS experiments, analyzed the data, and wrote the manuscript. VG-S analyzed the data and wrote the manuscript. IG-G performed the proteomics experiments. RS-L performed the proteomics experiments. MC-S performed the proteomics experiments and wrote the manuscript. AM-A performed the transcriptomics experiments. LV supervised and wrote the manuscript. JJ-N conceived and designed the experiments, supervised and wrote the manuscript.

## Conflict of Interest Statement

The authors declare that the research was conducted in the absence of any commercial or financial relationships that could be construed as a potential conflict of interest.

## Footnotes

1 ftp://ftp.uniprot.org/pub/databases/uniprot/uniref/uniref90/

2 http://www.plabipd.de/portal/mercator-sequence-annotation/

3 http://mzmine.github.io/

4 http://www.amdis.net/

5 http://chemdata.nist.gov/mass-spc/ms-search/

6 http://www.genome.jp/kegg/compound/

7 https://www.genome.jp/kegg/tool/map_pathway1.html

8 http://mapman.gabipd.org/web/guest/mapman/

9 http://www.uniprot.org/proteomes/UP000006548)

10 http://www.uniprot.org/proteomes/UP000059680

11 http://www.uniprot.org/proteomes/UP000006729

12 http://www.uniprot.org/proteomes/UP000030711

13 https://blast.ncbi.nlm.nih.gov/Blast.cgi

14 http://www.plabipd.de/portal/mercator-sequence-annotation/

15 https://mapman.gabipd.org/mapman-download/

16 https://www.arabidopsis.org/

17 http://www.genome.jp/kegg/compound/

18 https://www.plantcyc.org/

## Supplementary Material

FIGURE S1 Density histogram for proteins in the different biological processes of *Q. ilex* annotated transcriptome.

FIGURE S2 Density histogram for proteins in the different cellular components of *Q. ilex* annotated transcriptome.

FIGURE S3 Density histogram for proteins in the different molecular functions of *Q. ilex* annotated transcriptome.

FIGURE S4 Enzymes (transcript level and protein level) assigned to the glycolysis/gluconeogenesis.

TABLE S1 Metabolite features.

TABLE S2 GC-MS metabolomic data. Mean values of normalized peak areas, as well as SD (standard deviation) and CV% (percentage of coefficient of variation) were determined for replicates of the metabolite extract.

TABLE S3 KEGG pathways with metabolites, proteins, and transcripts.

TABLE S4 Comparison of KEGG pathways of different species.

TABLE S5 Bins of transcripts.

TABLE S6 Bins of proteins.

TABLE S7 Comparison of *in silico* proteomes.

TABLE S8 Shotgun LC-MS/MS proteomic data. Mean values of normalized peak areas, as well as SD (standard deviation) and CV% (percentage of coefficient of variation) were determined for replicates of protein extract (Jorge et al., 2005, 2006). The area values correspond to replicate 1 (0.6 μg of protein), replicate 2 (0.8 μg of protein), and replicate 3 (1 μg of protein).

TABLE S9 Enzymes (transcripts).

TABLE S10 Enzymes (proteins).

TABLE S11 Omics overview.
