# Supplementary figures and images for: A Multi-Omics Analysis Pipeline for the Metabolic Pathway Reconstruction in the Orphan Species Quercus ilex
Source: Front Plant Sci. 2018 Jul 11;9:935. doi: 10.3389/fpls.2018.00935 (PMC6050436; doi:10.3389/fpls.2018.00935)

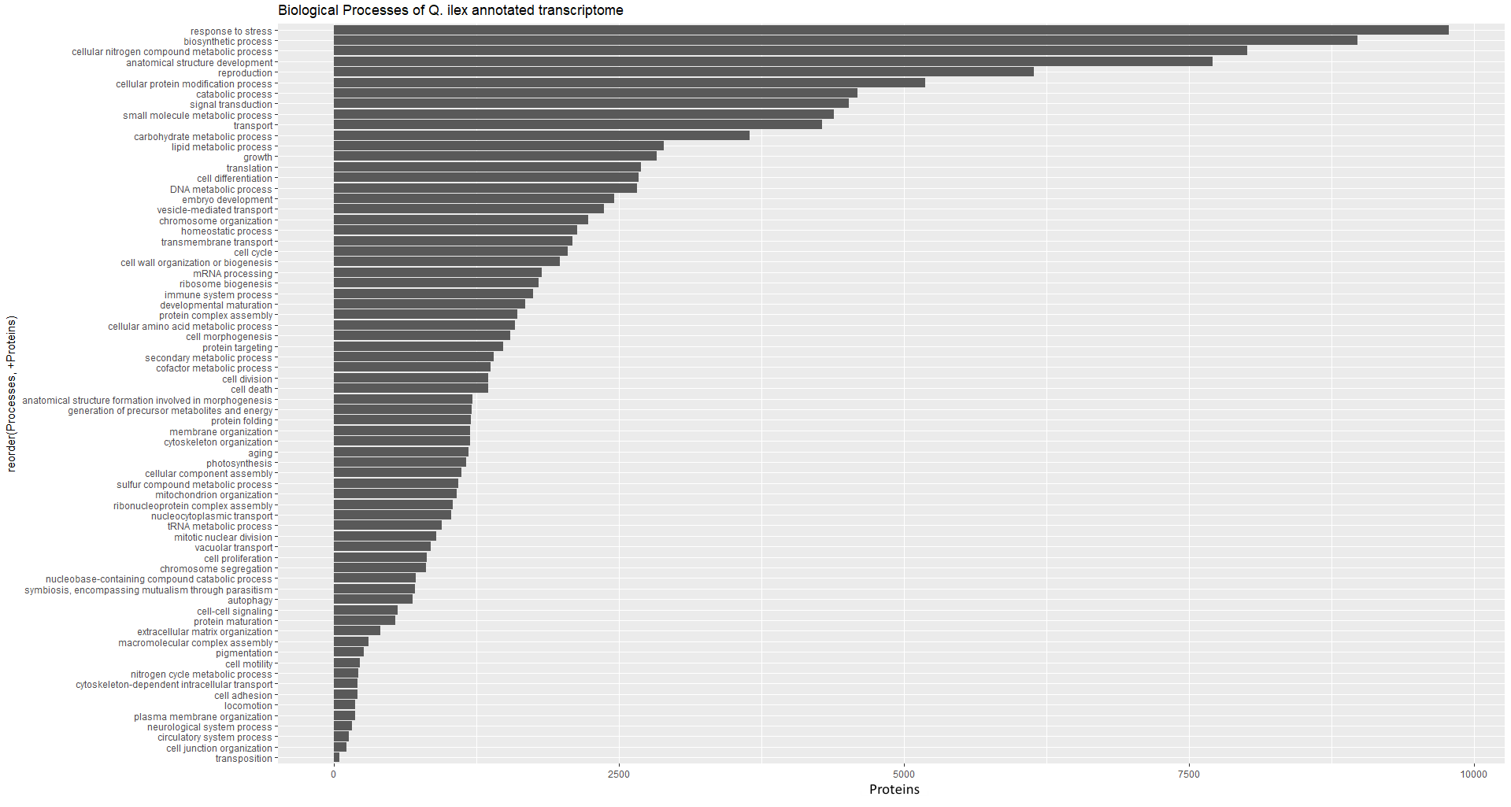

Supplement: FIGURE S1 — Density histogram for proteins in the different biological processes of Q. ilex annotated transcriptome. [file Image_1.TIF]

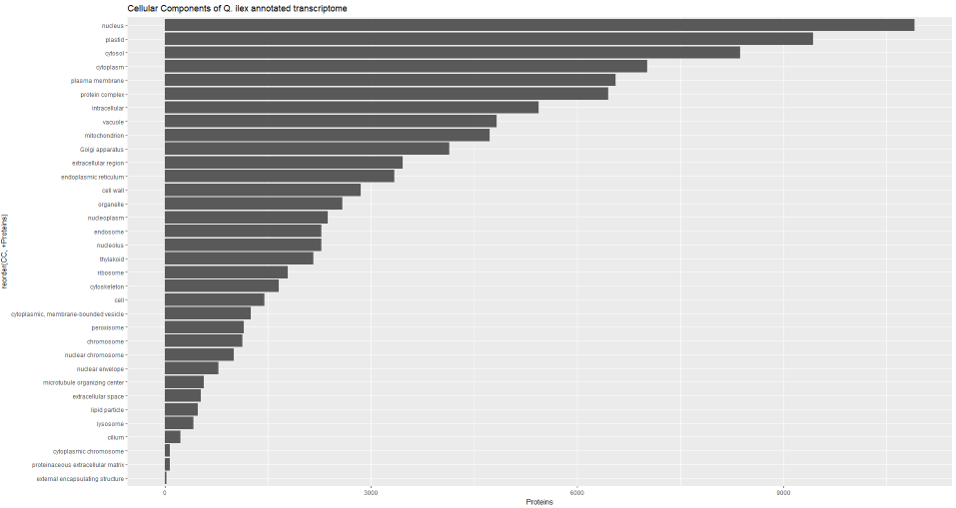

Supplement: FIGURE S2 — Density histogram for proteins in the different cellular components of Q. ilex annotated transcriptome. [file Image_2.TIF]

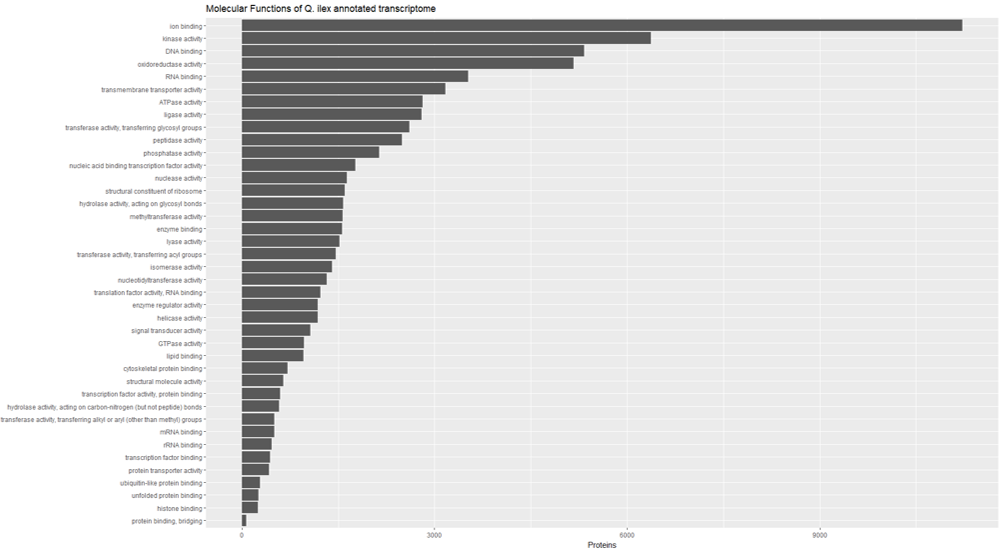

Supplement: FIGURE S3 — Density histogram for proteins in the different molecular functions of Q. ilex annotated transcriptome. [file Image_3.TIF]

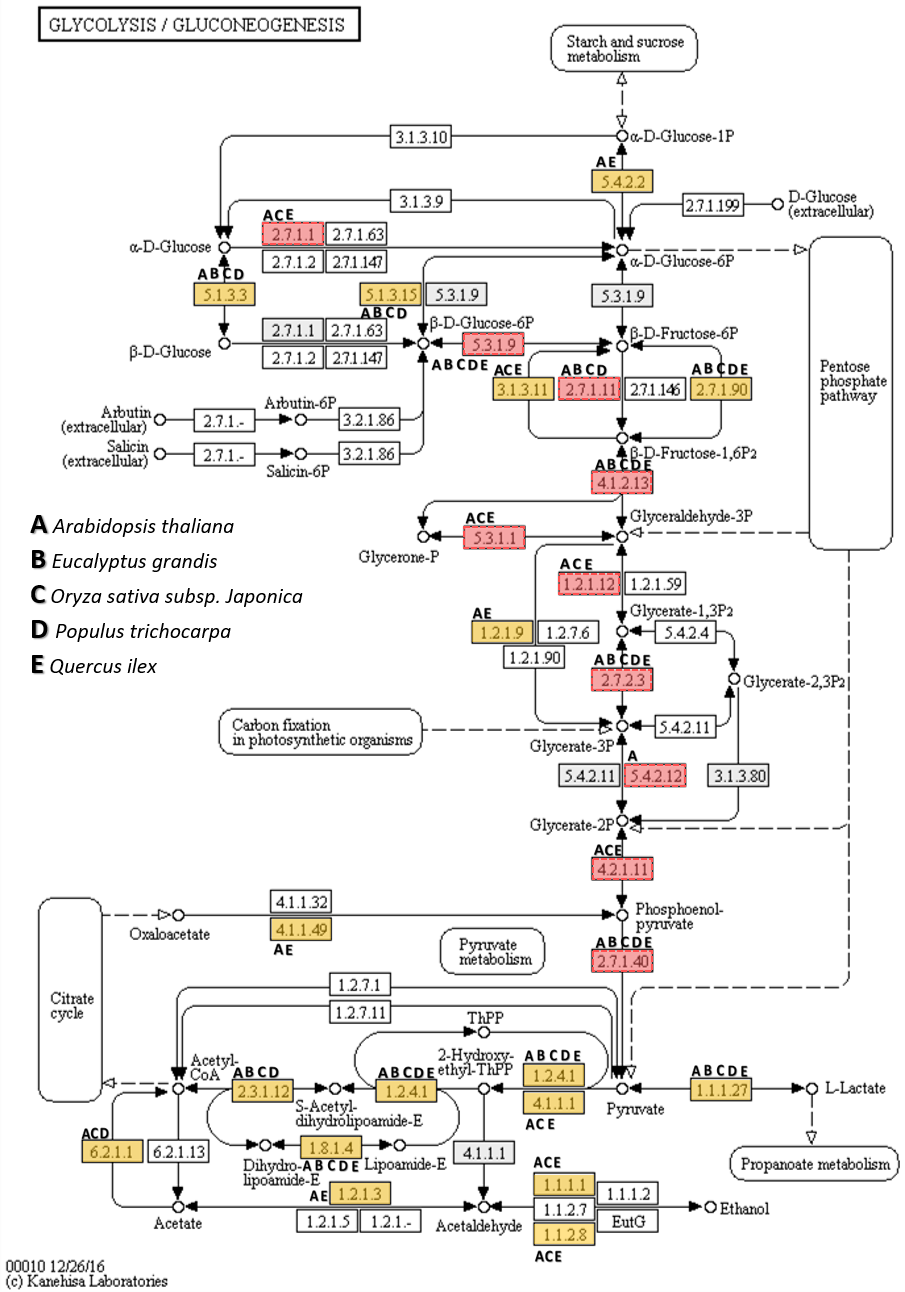

Supplement: FIGURE S4 — Enzymes (transcript level and protein level) assigned to the glycolysis/gluconeogenesis. [file Image_4.TIF]
